# Transcriptomic Analysis of *Paulownia* Infected by Paulownia Witches'-Broom *Phytoplasma*

**DOI:** 10.1371/journal.pone.0077217

**Published:** 2013-10-10

**Authors:** Hai-Qing Mou, Jie Lu, Shui-Fang Zhu, Cai-Li Lin, Guo-Zhong Tian, Xia Xu, Wen-Jun Zhao

**Affiliations:** 1 Institute of Plant Quarantine, Chinese Academy of Inspection and Quarantine, Beijing, China; 2 Institute of Forest Ecology, Environment and Protection, Chinese Academy of Forestry, Beijing, China; Virginia Tech, United States of America

## Abstract

Phytoplasmas are plant pathogenic bacteria that have no cell wall and are responsible for major crop losses throughout the world. *Phytoplasma*-infected plants show a variety of symptoms and the mechanisms they use to physiologically alter the host plants are of considerable interest, but poorly understood. In this study we undertook a detailed analysis of Paulownia infected by Paulownia witches’-broom (PaWB) *Phytoplasma* using high-throughput mRNA sequencing (RNA-Seq) and digital gene expression (DGE). RNA-Seq analysis identified 74,831 unigenes, which were subsequently used as reference sequences for DGE analysis of diseased and healthy Paulownia in field grown and tissue cultured plants. Our study revealed that dramatic changes occurred in the gene expression profile of Paulownia after PaWB *Phytoplasma* infection. Genes encoding key enzymes in cytokinin biosynthesis, such as isopentenyl diphosphate isomerase and isopentenyltransferase, were significantly induced in the infected Paulownia. Genes involved in cell wall biosynthesis and degradation were largely up-regulated and genes related to photosynthesis were down-regulated after PaWB *Phytoplasma* infection. Our systematic analysis provides comprehensive transcriptomic data about plants infected by *Phytoplasma*. This information will help further our understanding of the detailed interaction mechanisms between plants and *Phytoplasma*.

## Introduction

Phytoplasmas are specialized obligate bacteria at the phloem tissue of plant and are transmitted by insect vectors [1].. Over 700 plant diseases worldwide are associated with *Phytoplasma*, which have had a devastating effect on agricultural production [2,3]. For example, at least 50% of Florida’s estimated coconut palms and over 80% of Jamaica’s coconut palms have been killed by coconut lethal yellowing *Phytoplasma* during the last three decades [4]. 

Plants infected with *Phytoplasma* develop disease symptoms, such as witches’-broom, yellowing or reddening of leaves, short internodes, stunting and decline, phyllody, virescence, sterile flowers and necrosis [5]. The pathogenic mechanisms used by *Phytoplasma* are complex and interesting, but are largely unknown. Recently, it was reported that the expression levels of *PFG*, *PhGLO1* and *FBP7* genes, which are homeotic genes required for tissue development, were significantly down-regulated in the floral tissues of petunia (except the stamens) after *Phytoplasma* infection [6]. It has also been shown that ‘Bois noir’ *Phytoplasma* interacted with carbohydrate metabolism and down-regulated several photosynthetic genes in infected grapes, whereas defense genes, such as flavonoid metabolism-related and some pathogenesis related (PR) genes, were significantly up-regulated [7]. In Chardonnay grapes infected by ‘Bois noir’ *Phytoplasma*, serious inhibition of the whole photosynthetic chain and photosystem I activity, Calvin-cycle enzyme transcription, lipid metabolism and phenylpropanoid biosynthesis was observed. Moreover, the genes responsible for cell wall degradation were repressed in Chardonnay and Manzoni grape cultivars infected by ‘Bois noir’ *Phytoplasma*, whereas the genes involved in cell wall reinforcement were induced [8]. These studies have broadened our understanding of *Phytoplasma* pathogenesis and host defenses, but the physiological, biochemical and molecular mechanisms underlying disease symptom development are still poorly understood, because the inability of culturing phytoplasmas.

Paulownia witches’-broom (PaWB) *Phytoplasma* is the causative agent of paulownia (*Paulownia fortunei* (Seem.) Hemsl. x *P. tomentosa* (Thunb.) Steud.) witches’-broom disease. The typical symptoms of infected paulownia trees are proliferation of branches with very small yellowish leaves, followed by the dieback of branches, and then finally the tree dies; besides the symptoms consist of malformed flower buds with abnormally elongated calyxes. PaWB *Phytoplasma* is transmitted by leafhoppers (*Cicadella viridius*) as well as two types of stink bugs (*Halyomorpha mista* and *H. picus*), which has meant that the disease has spread throughout paulownia growing regions worldwide and has caused considerable damage to paulownia growth and wood production [9]. Understanding the responses of host plants to *Phytoplasma* infection is very important for developing efficient methods to control *Phytoplasma* diseases. In this study, we conducted a transcriptomic analysis of the paulownia plant responses to PaWB *Phytoplasma* infection using an RNA-Seq approach coupled with digital gene expression (DGE) analysis. After RNA-Seq analysis, over two billion bases of high-quality DNA sequences were generated using the Illumina HiSeq™ 2000 platform and 74,831 paulownia unigenes were finally obtained after sequence assembly. Furthermore, four DGE libraries (from two diseased samples and two healthy samples) were constructed in order to study the gene expression changes in paulownia after PaWB *Phytoplasma* infection. Our study investigated the genome-wide gene expression changes in paulownia after PaWB *Phytoplasma* infection and the results provide comprehensive information that improves our understanding of plant–*Phytoplasma* interactions.

## Materials and Methods

### Ethics statement

The paulownia plants in the field were grown in our institute, and our study had been permitted by our institute. Paulownia is the common tree species in China, and the paulownia withches’-broom disease is occurred widespread in north of China. Besides, the amount of sample collected from paulownia in the field was little; only one branch from each tree was collected. Therefore our field studies did not involve endangered or protected species.

### Plant growth and sample collection

In order to understand the responses of paulownia to PaWB *Phytoplasma* infection, we performed a transcriptomic analysis of samples from tissue cultured plants and field grown plants. In the tissue cultured group, healthy plants (TH) and plants infected with PaWB *Phytoplasma* (TD) were maintained in vitro on MS medium and then grown on a incubator at 25°C with a 16 h light and 8 h dark cycle. We also collected samples of leaves from diseased field grown plants (FD) and healthy plants (FH) in August 2011. For each sample, materials from three individuals were mixed together. 

### PCR tests for PaWB *Phytoplasma*

Total RNA was extracted from the midrib tissue using the cetyl trimethyl ammonium bromide (CTAB) method [10]. Direct PCR amplification was performed using R16F2n/R16R2 primers [11]. The PCR products were amplified, cloned and sequenced using an automated DNA sequencer (ABI Prism model 3730XL) and M13-47 and RV-M primers. The sequence was also subjected to virtual RFLP analysis using the *i*Phyclassifier online tool [12]. 

### RNA extraction

The healthy samples and PaWB *Phytoplasma*-infected samples, which had been confirmed by a PCR-test, were then used to prepare samples for RNA-Seq and DGE. For each sample, the total RNA was extracted from 3 g of tissue material using TRIzol® Reagent (Invitrogen), according to the manufacturer’s instructions, and then treated with DNase I (Invitrogen). The RNA concentration and integrity were analyzed using an Agilent 2100 Bioanalyzer (Agilent Technologies).

### Library construction and sequencing for RNA-seq

Equal quantities of total RNA from four samples were mixed. Poly(A)-containing mRNA was isolated using magnetic beads with oligo(dT) and then cut into short fragments. These short fragments were used as templates in order to synthesize the first-strand cDNA using reverse transcriptase and random hexamer-primers. The second-strand cDNA was synthesized using DNA polymerase I, dNTPs and RNase H. The double-stranded cDNA fragments were purified, end repaired, added to poly (A) and finally connected with sequencing adapters. After agarose gel electrophoresis, the suitable fragments were amplified through PCR to obtain the final library. The library was sequenced using Illumina HiSeq™ 2000 and the raw reads, generated by Solexa/Illumina sequencing, were submitted to the SRA database (NCBI), Accession No. SRA061384.

### Sequence assembly and analysis of RNA-seq

Clean reads were obtained for sequence assembly after removing reads containing adaptors, reads where more than 10% of the bases were unknown, and reads where more than half of the base quality values were less than 5. Transcriptome *de novo* assembly was carried out using SOAPdenovo [13]. First, the clean reads were combined with a specified length of overlap to form contigs. Then the pair-end reads were mapped back to the contigs so that contigs from the same transcript could be detected, as well as the distances between these contigs. Second, the contigs were connected to make scaffolds, using N to represent unknown sequences between each two contigs. Next, the paired-end reads were used to gap-fill the scaffolds in order to get sequences with the least number of Ns and sequences that could not to be extended at either end, which were defined as unigenes. 

The unigenes were analyzed by searching protein databases, such as: nr, Swiss-Prot, KEGG and COG using the blastx (evalue < 0.00001) program [14]. If the results from the different databases conflicted with each other, then the priority order: nr, Swiss-Prot, KEGG and COG, was followed when deciding the unigene sequence direction. The results from blast analysis were used to extract CDSs from unigene sequences and translate them into peptide sequences. Unigenes with no hit in blast were analyzed using ESTScan software [15] to predict their coding regions and decide their sequence direction and those with nr annotations were further analyzed with Blast2go [16] to get GO annotations. Unigenes with GO annotations were classified according to the GO functional classification using WEGO software [17] in order to understand the distribution of gene functions in paulownia at the macro level.

### Library construction and sequencing for digital gene expression (DGE)

Four tag libraries were constructed in parallel. Sequence tags were prepared using the Illumina Gene Expression Sample Prep Kit according to the manufacturer’s instructions. Magnetic beads with oligo (dT) were used to purify poly (A)-containing mRNA and an oligo-dT primer was used to synthesize double-stranded cDNA. The bead-bound cDNAs were then digested by endonuclease *Nla*III, which recognizes and cuts CATG sites and generates sticky 5′ ends. The fragments that had been cut off were washed away and the bead-bound fragments were ligated to the Illumina adaptor 1 at the sticky 5′ end. The bead-bound fragments that contained adaptor 1 were then digested by *Mme*I, which cuts at 17 bp downstream of the CATG site. After that, the bead-bound fragments without adaptor 1 were removed by magnetic bead precipitation and the tags with adaptor 1 were purified and ligated to Illumina adaptor 2 at the 3′ end of the tag. After 15 cycles of linear PCR amplification, the 95 bp fragments were purified using 6% TBE polyacrylamide gel electrophoresis to obtain the final tag libraries. After denaturation, the single-stranded DNAs were fixed onto the Illumina Sequencing Chip (flowcell). Four types of nucleotides, labeled with four different colors, were sequenced using Illumina HiSeq™ 2000 and the sequencing by synthesis (SBS) method. Millions of raw reads with sequencing lengths of 35 bp were generated in each tunnel. The raw tag data were deposited in the GEO database (NCBI), Accession Nos. GSM1032231 (TH), GSM1032232 (TD), GSM1032233 (FH), GSM1032234 (FD).

### Data analysis of DGE profiling

The adaptors, empty tags (no tag sequence between the adaptors), low quality tags (tags with unknown nucleotide ‘‘N’’) and tags with a copy number of one were removed from the raw data in order to obtain 21 bp clean tags. All the clean tags were mapped to the transcriptome reference database generated by RNA-Seq. Both the sense and complementary antisense sequences were used in the mapping process so that mapping events on both strands could be monitored. 

The number of unambiguous tags corresponding to each gene was calculated and normalized to TPM (number of transcript per million clean tags) so that the expression of different genes could be analyzed. A rigorous algorithm was developed, based on the method described by Audic and Claverie, in order to identify the differentially expressed genes between the *Phytoplasma*-infected sample and healthy sample [18]. The FDR (false discovery rate) was used to determine the *P*-value threshold. An FDR ≤ 0.001 and an absolute value for the log 2 ratio of ≥ 1 were selected as the threshold in order to judge whether the change in gene expression was significant. Genes that showed significant expression changes were used for KEGG pathway enrichment analysis. The formula for calculating the enriched *P*-value was:

$$\text{P}=1-\sum_{i=0}^{m-1}\frac{\left(\begin{array}{c} M\\ i \end{array}\right)\left(\begin{array}{c} N-M\\ n-i \end{array}\right)}{\left(\begin{array}{c} N\\ n \end{array}\right)}$$

where *N* is the number of genes with a KEGG annotation, *n* is the number of differentially expressed genes in *N*, *M* is the number of genes annotated to specific pathways and *m* is the number of differentially expressed genes in *M*. A *P*-value of 0.05 was selected as the significance threshold for enrichment of a gene set. 

### Quantitative real-time PCR (qRT-PCR) validation

Total RNA (5 µg) from each sample was reverse transcribed with the SuperScript III First-Strand Synthesis System for RT-PCR (Invitrogen), according to the manufacturer’s protocols. Samples of cDNA were used for quantitative real-time PCR using the ABI Prism 7900 HT real-time PCR system (Applied Biosystems) with SYBR Premix ExTaq (Applied Biosystems). The real-time PCR primers were designed using Primer Premier 5. Real-time RT-PCR analysis was performed three times for each RNA sample extracted. The cycle threshold (CT) value for each gene was normalized with the CT value of the actin gene using RQ Manager software (Applied Biosystems). Standard curves for each gene expression were generated in each experiment. 

## Results

### Detection of PaWB *Phytoplasma* in paulownia showing disease symptoms

**Figure 1:**
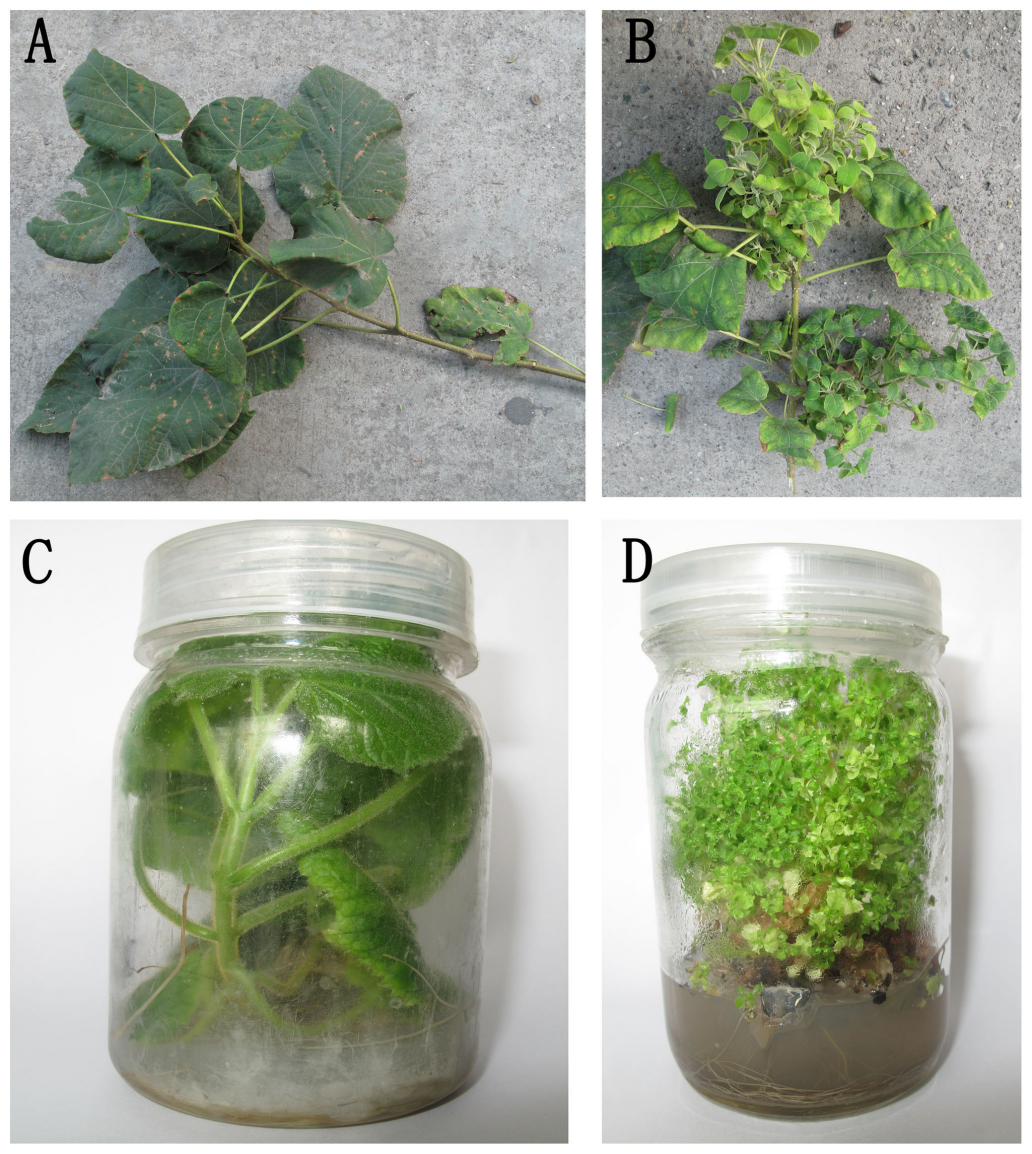
Symptoms shown by paulownia when infected with PaWB *Phytoplasma*. (A) a healthy branch of paulownia in the field; (B) symptoms shown by a branch infected with PaWB *Phytoplasma* in the field; (C) a healthy branch of paulownia grown in the culture medium and (D) symptoms shown by a branch infected with PaWB *Phytoplasma* when grown in the culture medium.

After PaWB *Phytoplasma* infection, the paulownia plants mainly showed typical disease symptoms, such as witches’-broom, yellowing of leaves, smaller leaves, short internodes, phyllody and sterile flowers (Figure 1). Branches showing disease symptoms always withered and died in the following year. The 1.24 kb fragments of 16S rDNA from PaWB *Phytoplasma* could be detected in the midribs of symptomatic plants, but not from the symptomless plants. The amplified 16S rDNA fragment shared a 100% nucleotide sequence identity with previously reported PaWB *Phytoplasma* (GenBank Accession No.HM146078). Using virtual RFLP analysis, we confirmed that the PaWB *Phytoplasma* in our study belonged to the 16SrI-B subgroup (data not shown). 

### Sequence assembly and coding sequences

**Figure 2:**
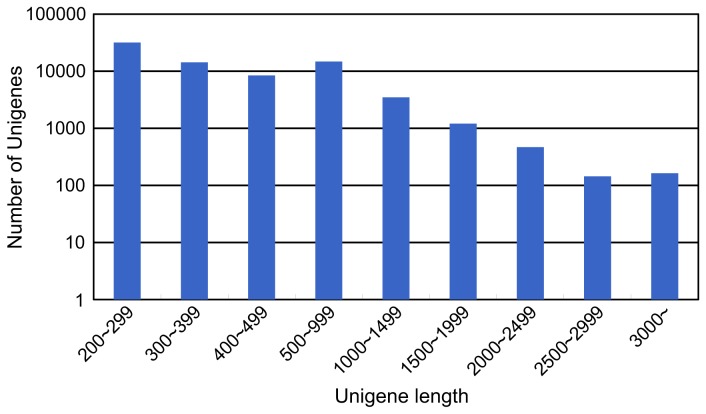
Distribution of unigene sizes.

Since the genome sequence for paulownia was not available, we carried out an RNA-Seq analysis in order to obtain the mRNA sequences for paulownia. Equal quantities of total RNA from the four samples (TH, TD, FH and FD) were mixed and used in the library construction for Illumina HiSeq 2000 sequencing. A total of 27,369,188 clean reads (2,463,226,920 nucleotides) were generated by Illumina deep sequencing and the Q20 percentage (proportion of nucleotides with a quality value larger than 20) for the data was 95.25%. These clean tags were assembled into 508,537 contigs and then connected to 133,983 scaffolds. After gap filling using paired-end reads, the scaffolds were assembled into 74,831 unigenes. The unigene size distribution is shown in Figure 2. The mean length of the unigenes was 464 bp.

Blastx and ESTscan analysis indicated that 48,712 unigenes had reliable coding sequences (65.1% of all unigenes). Blastx analysis (E-value < 0.00001) showed that 47,801, 30,998, 21,875 and 13,011 unigenes produced BLAST results against the non-redundant (nr, NCBI) protein database, the Swiss-Prot database, the Kyoto Encyclopedia of Genes and Genomes (KEGG) database and the Clusters of Orthologous Groups of proteins (COG, NCBI) database, respectively. Blast2go analysis of the unigenes with nr annotations further revealed that 20,603 unigenes had Gene Ontology (GO) annotations. Unigenes without blast hits were analyzed using ESTscan and the coding sequences for 1,705 unigenes were predicted and translated into peptide sequences.

### Unigene function and pathway annotations

**Figure 3:**
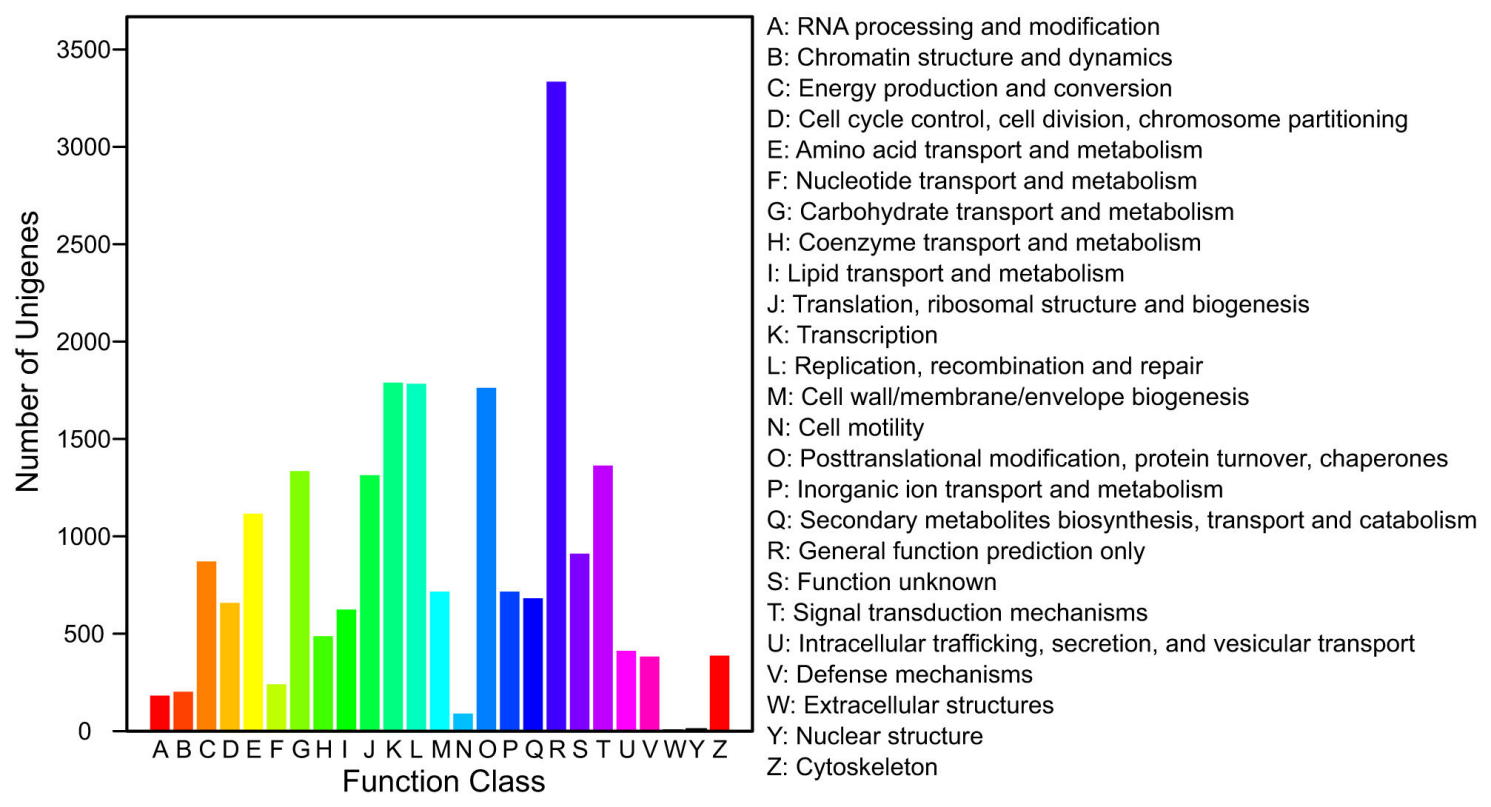
Unigene COG annotations. Unigenes aligned to the COG database can be classified functionally into 25 molecular families.

**Figure 4:**
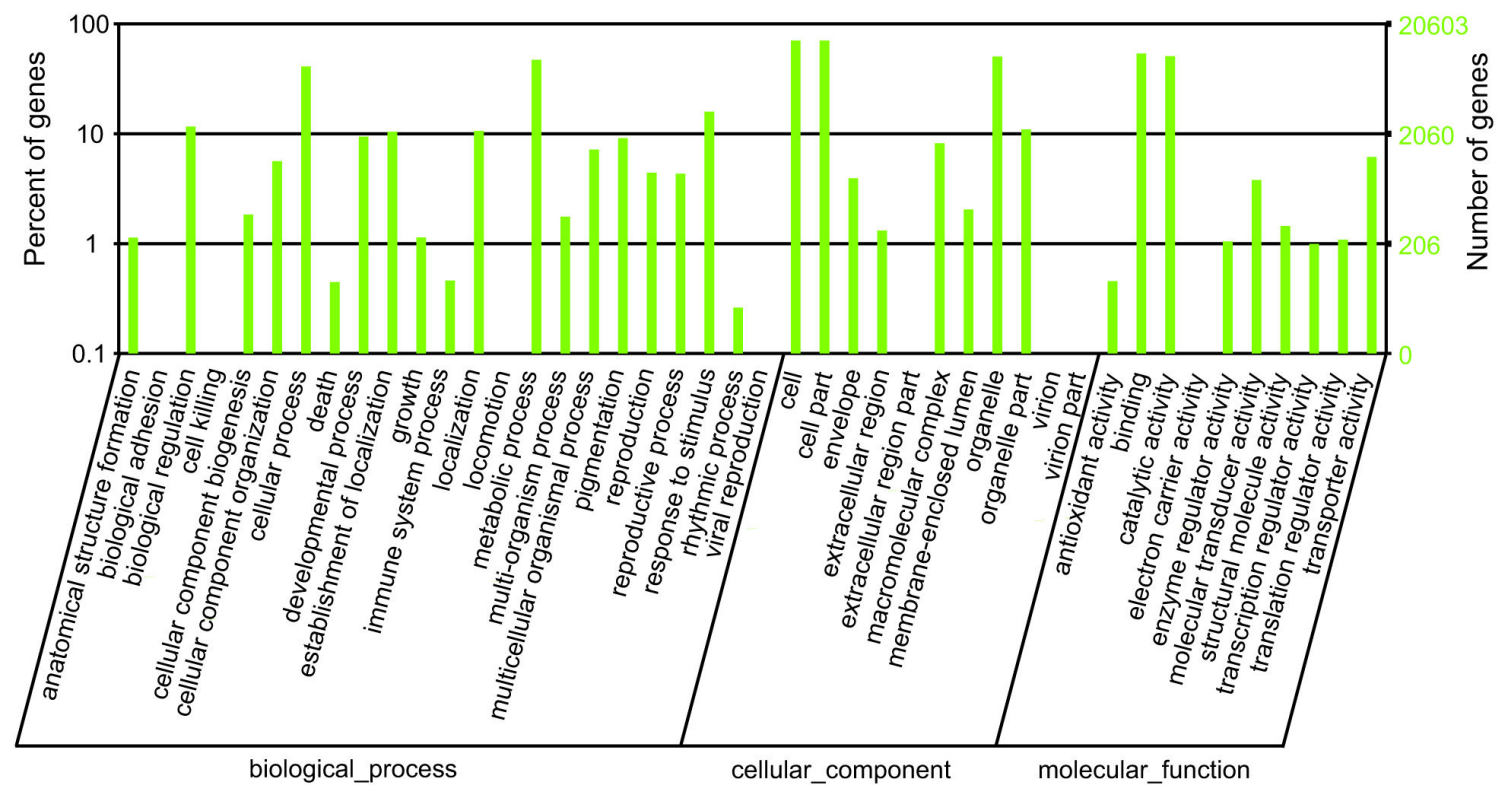
GO annotation of unigenes. Best hits were aligned to the GO database and transcripts were assigned to at least one GO term. Most unigenes were grouped into three major functional categories, namely biological processes, cellular components, and molecular functions.

The sequences with blast hits were further analyzed to get their COG functional annotations, their GO functional annotations and their KEGG pathway annotations. The COG analysis indicated that 13,011 unigenes had COG annotations and could be grouped into 25 clusters (Figure 3). The GO analysis showed that 20,603 unigenes had GO annotations that could be grouped into 44 classes in the three ontologies, including growth, development, cell death, metabolism and transcription regulation (Figure 4). The KEGG pathway analysis revealed that 21,875 of the unigenes could be grouped into 119 known pathways. The three pathways that contained the largest numbers of genes were the metabolic pathways, the biosynthesis of secondary metabolites and plant-pathogen interaction pathways (Table 1).

**Table 1:** Top 20 unigene enriched KEGG pathways.

Pathway	Unigenes with pathway annotation	Pathway ID
Metabolic pathways	5009 (22.9%)	ko01100
Biosynthesis of secondary metabolites	2712 (12.4%)	ko01110
Plant-pathogen interaction	1832 (8.37%)	ko04626
Spliceosome	1064 (4.86%)	ko03040
Protein processing in endoplasmic reticulum	778 (3.56%)	ko04141
Starch and sucrose metabolism	747 (3.41%)	ko00500
Phenylpropanoid biosynthesis	512 (2.34%)	ko00940
Ribosome	485 (2.22%)	ko03010
Ubiquitin mediated proteolysis	485 (2.22%)	ko04120
Purine metabolism	450 (2.06%)	ko00230
Glycolysis / Gluconeogenesis	410 (1.87%)	ko00010
Endocytosis	390 (1.78%)	ko04144
Pyrimidine metabolism	379 (1.73%)	ko00240
Amino sugar and nucleotide sugar metabolism	353 (1.61%)	ko00520
Cysteine and methionine metabolism	344 (1.57%)	ko00270
Stilbenoid, diarylheptanoid and gingerol biosynthesis	337 (1.54%)	ko00945
Oxidative phosphorylation	322 (1.47%)	ko00190
Limonene and pinene degradation	310 (1.42%)	ko00903
RNA degradation	306 (1.4%)	ko03018
Pyruvate metabolism	298 (1.36%)	ko00620

### Digital gene expression (DGE) profiles of the different samples

To reveal the biological processes influenced by PaWB *Phytoplasma* infection, we analyzed the gene expression of the four samples (TH, TD, FH and FD) via DGE profiling. DGE libraries for the four samples were constructed and sequenced in parallel. More than three million raw tags were generated for each DGE library and over 90% of the raw tags in each library were clean tags (Figure S1). To evaluate the normality of the whole data, the distribution of clean tag expressions were analyzed. In our analysis, total clean tags represent the sum of all the clean tag numbers and distinct clean tags represent all types of clean tags. In each library, about 60% of the total clean tags were tags with copy numbers of more than 100, which only made up 3–4% of the distinct clean tags. In contrast, tags with copy numbers between 2 and 5 accounted for 7% of total clean tags, but accounted for 60% of the distinct clean tags. These results were in accordance with the rule that a small number of mRNA categories are highly expressed, while the majority of categories have low expression levels, which indicated that the DGE data was normally distributed (Figure S2).

The 56,924 unigenes (76.07% of all unigenes) with CATG sites were used as reference genes, of which 25,446 unigenes were tag-mapped genes. Among the distinct clean tags from the four libraries, 152,235 distinct clean tags were mapped to the reference genes and the unambiguous tags made up 151,260 of the distinct clean tags, which accounted for 99.36% of all reference tags. The numbers of distinct clean tags in each library ranged from 135,500 to 154,300, while distinct clean tags mapping to unigenes ranged from 57,281 to 74,937, or 40.56% to 49.84%, respectively. A summary of the DGE tag analysis is shown in Table 2.

**Table 2:** Tag analysis results.

Summary	TD	TH	FD	FH
Raw tag	3,712,343	3,510,991	3,687,700	3,680,236
Distinct raw tag	357,270	364,082	368,807	327,753
Clean tag	3,493,642	3,287,059	3,465,097	3,476,593
Distinct clean tag	147,844	150,356	154,300	135,500
Clean tag/raw tag	94.11%	93.62%	93.96%	94.47%
All tag mapping to gene	1,555,312	1,427,939	1,465,155	1,313,648
All tag mapping to gene (% of clean tag)	44.52%	43.44%	42.28%	37.79%
Distinct tag mapping to gene	62,651	74,937	62,589	57,281
Distinct tag mapping to gene (% of clean tag)	42.38%	49.84%	40.56%	42.27%
Unambiguous tag mapping to gene	1,530,564	1,413,537	1,443,754	1,289,951
Unambiguous tag mapping to gene (% of clean tag)	43.81%	43.00%	41.67%	37.10%
Distinct unambiguous tag mapping to gene	62,170	74,314	62,079	56,810
Distinct unambiguous tag mapping to gene (% of clean tag)	42.05%	49.43%	40.23%	41.93%
All tag-mapped genes	22,539	24,499	23,146	22,022
All tag-mapped genes (% of ref genes)	30.12%	32.74%	30.93%	29.43%
Unambiguous tag-mapped genes	22,211	24,167	22,804	21,705
Unambiguous tag-mapped genes (% of ref genes)	29.68%	32.30%	30.47%	29.01%
Unknown tag	1,938,330	1,859,120	1,999,942	2,162,945
Unknown tag (% of clean tag)	55.48%	56.56%	57.72%	62.21%
Distinct unknown tag	85,193	75,419	91,711	78,219
Distinct unknown tag (% of clean tag)	57.62%	50.16%	59.44%	57.73%

### Gene expression changes after PaWB infection

**Figure 5:**
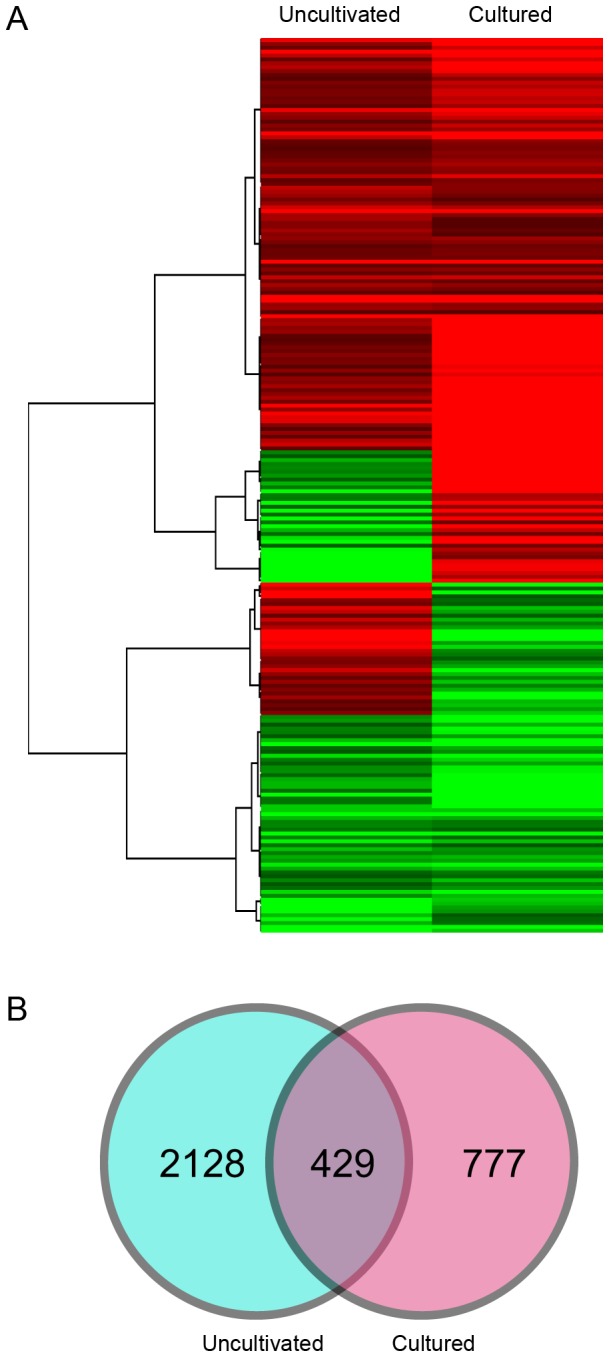
Relationship between DEGs in the two groups (A) and cluster analysis of the common DEGs found in the two groups (B).

In the field grown group, 2,557 genes showing significant differential expressions were identified in the diseased sample (FD) compared to healthy sample (FH), of which, 1,271 were up-regulated genes and 1,286 were down-regulated genes (Figure 5). According to the KEGG pathway analysis, there were 25 pathways that contained significantly enriched gene sets. Twenty-three pathways were related to metabolism, one pathway (Peroxisome) was related to cellular processes, and one pathway (Ribosome) was related to genetic information processing. Five metabolic pathways were correlated to lipid metabolism, five pathways to terpenoid and polyketide metabolism and five pathways were correlated to the biosynthesis of other secondary metabolites (Table S1).

In the tissue cultured group, 1,206 genes with significant differential expressions were identified in the diseased sample (TD) compared to the healthy sample (TH), of which, 769 were up-regulated and 437 were down-regulated (Figure 5). According to the KEGG pathway analysis, 23 pathways showed significantly enriched gene sets. Among them, 21 pathways were related to metabolism, one (Peroxisome) was related to cellular processes and one pathway (Ribosome) was related to genetic information processing. Five metabolic pathways were correlated to lipid metabolism, three pathways to the biosynthesis of other secondary metabolites and three pathways were correlated to terpenoid and polyketide metabolism (Table S2). 

### Biological processes influenced by PaWB infection

There were 19 common pathways that were significantly enriched in both two groups, according to the KEGG pathway analysis (Table 3). These results suggested that *Phytoplasma* infection significantly suppressed the photosynthesis process and disturbed the metabolic processes of many secondary metabolites, such as plant hormones and phenylpropanoids, which may result in the macroscopic symptoms exhibited by the paulownia plants. To further investigate the biological processes affected by PaWB infection, the DEGs that were common to both groups were analyzed. A total of 429 unigenes were identified as common DEGs between the field grown group and the tissue cultured group (Figure 5). BLAST analysis revealed that only 100 of them had KEGG function annotations and further analysis indicated that these DEGs were mostly correlated to biological processes, such as energy metabolism, translation, carbohydrate metabolism, hormone biosynthesis and transduction, defense reactions and transport (Table 4). 

**Table 3:** KEGG pathways that were common to both groups.

	**FH&FD**		**TH&TD**	
**Pathway**	**Up-regulated**	**Down-regulated**	**Up-regulated**	**Down-regulated**
Photosynthesis	0	20	0	10
alpha-Linolenic acid metabolism	4	17	6	6
Linoleic acid metabolism	3	12	5	6
Biosynthesis of unsaturated fatty acids	3	16	3	5
Fatty acid metabolism	3	17	5	5
Carotenoid biosynthesis	4	10	4	3
Terpenoid backbone biosynthesis	5	4	3	4
Limonene and pinene degradation	4	18	8	5
Flavonoid biosynthesis	14	8	7	5
Phenylpropanoid biosynthesis	24	16	12	4
Stilbenoid, diarylheptanoid and gingerol biosynthesis	6	18	7	4
Metabolism of xenobiotics by cytochrome P450	1	19	5	4
Arginine and proline metabolism	5	9	4	5
Glutathione metabolism	4	11	6	2
Biosynthesis of phenylpropanoids	24	29	14	11
Biosynthesis of plant hormones	23	48	16	15
Peroxisome	5	15	3	7
Ribosome	12	15	5	10

**Table 4:** KEGG-annotated DEGs that were common to both groups.

	**Fold change (log_2_R)**			
**Unigene ID**	**TD/ TH**	**FD/ FH**	**Definition (KO ID)**	**Pathway (Pathway ID)**
**Energy metabolism**				
Unigene23994	-1.58	-2.31	Photosystem I core protein PsaB (K02690)	Photosynthesis (ko00195)
Unigene41021	-1.34	-1.12	Photosystem I subunit II PsaD (K02692)	Photosynthesis (ko00195)
Unigene57786	-2.22	-4.11	Photosystem II core protein PsbA (K02703)	Photosynthesis (ko00195)
Unigene70434	-3.45	-1.77	Photosystem II oxygen-evolving enhancer protein PsbP (K02717)	Photosynthesis (ko00195)
Unigene73155	-1.26	-1.75	Photosystem II oxygen-evolving enhancer protein PsbQ(K08901)	Photosynthesis (ko00195)
Unigene67411	-2.07	-1.29	Photosystem II 10kDa protein PsbR (K03541)	Photosynthesis (ko00195)
Unigene40507	-1.22	-1.99	Photosystem II 22kDa protein PsbS (K03542)	Photosynthesis (ko00195)
Unigene27161	-1.50	-1.61	F-type ATPase subunit alpha [EC:3.6.3.14] (K02111)	Photosynthesis (ko00195)
Unigene72265	-2.31	-1.75	F-type ATPase subunit delta [EC:3.6.3.14] (K02113)	Photosynthesis (ko00195)
Unigene57813	-1.05	-1.87	light-harvesting complex I Chlorophyll a/b binding protein LHCA2 (K08908)	Photosynthesis - antenna proteins (ko00196)
Unigene65637	-3.42	-1.22	light-harvesting complex I Chlorophyll a/b binding protein LHCA5 (K08911)	Photosynthesis - antenna proteins (ko00196)
Unigene64828	1.12	1.85	V-type H+-transporting ATPase subunit E [EC:3.6.3.14] (K02150)	Oxidative phosphorylation (ko00190)
Unigene13672	1.59	1.02	NADH dehydrogenase 1 alpha subcomplex 2 [EC:1.6.5.3 1.6.99.3] (K03946)	Oxidative phosphorylation (ko00190)
Unigene73041	1.63	8.93	Carbonic anhydrase [EC:4.2.1.1] (K01674)	Nitrogen metabolism (ko00910)
**Translation**				
Unigene60861	1.38	1.02	Prolyl-tRNA synthetase [EC:6.1.1.15] (K01881)	Aminoacyl-tRNA biosynthesis (ko00970)
Unigene50004	1.32	1.34	Asparaginyl-tRNA synthetase [EC:6.1.1.22] (K01893)	Aminoacyl-tRNA biosynthesis (ko00970)
Unigene44862	1.05	2.70	60S large subunit ribosomal protein L12e (K02870)	Ribosome (ko03010)
Unigene12998	2.41	3.49	30S small subunit ribosomal protein S17 (K02961)	Ribosome (ko03010)
Unigene53304	1.18	2.44	50S ribosomal protein L33-like (K02913)	Ribosome (ko03010)
Unigene77	-1.67	-2.85	50S ribosomal protein L1, chloroplastic-like (K02863)	Ribosome (ko03010)
Unigene52586	-3.66	-2.94	50S ribosomal protein L15, chloroplastic-like (K02876)	Ribosome (ko03010)
Unigene24837	-1.79	-1.89	50S ribosomal protein L27, chloroplastic-like (K02899)	Ribosome (ko03010)
Unigene60918	-1.35	-1.01	50S ribosomal protein L29, chloroplastic-like (K02904)	Ribosome (ko03010)
Unigene68812	-4.01	-2.38	31 kDa ribonucleoprotein, chloroplastic-like (K02965)	Ribosome (ko03010)
Unigene1219	-1.55	-2.99	Elongation factor EF-G, chloroplastic-like (K02355)	Ribosome (ko03010)
Unigene62803	-1.04	-1.47	Elongation factor EF-Tu, chloroplastic-like (K02358)	Ribosome (ko03010)
Unigene37795	-11.13	-2.41	40s small subunit ribosomal protein S12e (K02951)	Ribosome (ko03010)
Unigene74177	-1.02	-1.24	Translation initiation factor IF-2 unclassified subunit (K03243)	RNA transport (ko03013 )
**Carbohydrate metabolism**				
Unigene65685	1.62	2.38	UDP-glucose 4-epimerase [EC:5.1.3.2] (K01784)	Galactose metabolism (ko00052)
Unigene68516	1.98	1.62	alpha-galactosidase [EC:3.2.1.22] (K07407)	Galactose metabolism (ko00052)
Unigene23754	1.39	9.79	Inositol oxygenase [EC:1.13.99.1] (K00469)	Inositol phosphate metabolism (ko00562)
Unigene35494	1.36	1.21	Cellulose synthase A [EC:2.4.1.12] (K10999)	
Unigene112	1.69	4.37	Cellulose synthase A [EC:2.4.1.12] (K10999)	
Unigene36507	1.24	2.42	Pectinesterase [EC:3.1.1.11] (K01051)	Starch and sucrose metabolism (ko00500)
Unigene67084	1.70	4.34	Pectinesterase [EC:3.1.1.11] (K01051)	Starch and sucrose metabolism (ko00500)
Unigene66203	1.81	9.23	Beta-glucosidase [EC:3.2.1.21] (K01188)	Starch and sucrose metabolism (ko00500)
Unigene74438	-2.20	-1.06	Fructokinase [EC:2.7.1.4] (K00847)	Fructose and mannose metabolism (ko00051)
**Hormone related**				
Unigene68742	1.63	12.21	Phospholipase A2 [EC:3.1.1.4] (K01047)	alpha-Linolenic acid metabolism (ko00592)
Unigene6332	1.14	1.42	Enoyl-CoA hydratase/ 3-hydroxyacyl-CoA dehydrogenase [EC:4.2.1.17 1.1.1.35 1.1.1.211] (K10527)	alpha-Linolenic acid metabolism (ko00592)
Unigene10488	1.32	3.03	Farnesyl diphosphate synthase [EC:2.5.1.1 2.5.1.10] (K00787)	Terpenoid backbone biosynthesis (ko00900)
Unigene4457	1.39	1.69	Xanthoxin dehydrogenase [EC:1.1.1.288] (K09841)	Carotenoid biosynthesis (ko00906)
Unigene1276	1.05	2.82	Isopentenyl-diphosphate delta-isomerase [EC:5.3.3.2] (K01823)	Terpenoid backbone biosynthesis (ko00900)
Unigene22539	1.00	6.43	adenylate isopentenyltransferase (K10760)	Zeatin biosynthesis (ko00908)
Unigene554	7.43	8.01	adenylate isopentenyltransferase (K10760)	Zeatin biosynthesis (ko00908)
Unigene73098	-2.90	-2.31	Cytokinin trans-hydroxylase, CYP735A (K10717)	Zeatin biosynthesis (ko00908)
Unigene26784	1.09	1.59	phytochrome-interacting factor 3 (K12126)	Plant hormone signal transduction (ko04075)
Unigene74076	1.78	1.63	auxin influx carrier (AUX1 LAX family) (K13946)	Plant hormone signal transduction (ko04075)
Unigene70471	1.85	1.72	Delta24-sterol reductase [EC:1.3.1.72] (K09828)	Steroid biosynthesis (ko00100)
Unigene28278	2.48	2.05	Phospholipase C [EC:3.1.4.3] (K01114)	Ether lipid metabolism (ko00565)
**Defense related**				
Unigene25741	1.49	2.35	Serine/threonine-protein kinase PBS1 [EC:2.7.11.1] (K13430)	Plant-pathogen interaction (ko04626)
Unigene567	1.42	9.42	Serine/threonine-protein kinase PBS1 [EC:2.7.11.1] (K13430)	Plant-pathogen interaction (ko04626)
Unigene66077	2.47	9.36	LRR receptor-like serine/ threonine-protein kinase [EC:2.7.11.1] (K13420)	Plant-pathogen interaction (ko04626)
Unigene22762	1.13	1.29	Guanine nucleotide-exchange factor (K13462)	Plant-pathogen interaction (ko04626)
Unigene13928	1.03	1.45	L-ascorbate peroxidase [EC:1.11.1.11] (K00434)	Ascorbate and aldarate metabolism (ko00053)
Unigene814	-1.64	-2.20	(S)-2-hydroxy-acid oxidase [EC:1.1.3.15] (K11517)	Peroxisome (ko04146)
Unigene71586	-2.66	-4.26	Nitric-oxide synthase, plant [EC:1.14.13.39] (K13427)	Plant-pathogen interaction (ko04626)
**Transport**				
Unigene26611	-1.57	-1.38	Preprotein translocase subunit SecA (K03070)	Protein export (ko03060)
Unigene15161	1.20	1.54	Preprotein translocase subunit YidC (K03217)	Protein export (ko03060)
Unigene67494	1.23	1.69	Calreticulin (K08057)	Protein processing in endoplasmic reticulum (ko04141)
Unigene49593	1.73	1.39	Syntaxin 1B/2/3 (K08486)	SNARE interactions in vesicular transport (ko04130)
Unigene24327	1.70	1.89	ATP-binding cassette, subfamily B (MDR/TAP), member 1 (K05658)	ABC transporters (ko02010)
Unigene11045	1.85	10.42	ATP-binding cassette, subfamily B (MDR/TAP), member 1 (K05658)	ABC transporters (ko02010)
**Others**				
Unigene71221	-1.70	-2.87	Lipoxygenase [EC:1.13.11.12] (K00454)	alpha-Linolenic acid metabolism (ko00592)
Unigene66023	1.16	12.11	Cinnamoyl-CoA reductase [EC:1.2.1.44] (K09753)	Phenylpropanoid biosynthesis (ko00940)
Unigene66508	1.31	1.59	Cinnamyl-alcohol dehydrogenase [EC:1.1.1.195] (K00083)	Phenylpropanoid biosynthesis (ko00940)
Unigene27448	1.16	4.72	Cinnamyl-alcohol dehydrogenase [EC:1.1.1.195] (K00083)	Phenylpropanoid biosynthesis (ko00940)
Unigene61973	1.39	1.78	Fatty acyl-ACP thioesterase B [EC:3.1.2.14 3.1.2.-] (K10781)	Fatty acid biosynthesis (ko00061)
Unigene70702	-1.58	-1.43	Riboflavin kinase [EC:2.7.1.26] (K00861)	Riboflavin metabolism (ko00740)
Unigene58109	-2.18	-6.54	Cytochrome P450, family 3, subfamily A [EC:1.14.14.1] (K07424)	Steroid biosynthesis (ko00100)
Unigene69626	1.28	1.44	Cytochrome P450, family 72, subfamily A, polypeptide 1 (secologanin synthase) [EC:1.3.3.9] (K13400)	Monoterpenoid biosynthesis (ko00902)
Unigene27145	2.05	1.55	Gamma-glutamyltranspeptidase [EC:2.3.2.2] (K00681)	Glutathione metabolism (ko00480)
Unigene44406	1.46	2.49	Bifunctional dihydroflavonol 4-reductase/flavanone 4-reductase [EC:1.1.1.219] (K13082)	Flavonoid biosynthesis (ko00941)
Unigene60959	1.61	2.97	Tyrosine decarboxylase [EC:4.1.1.25] (K01592)	Tyrosine metabolism (ko00350)
Unigene63956	-2.63	-3.45	Hydroxypyruvate reductase 2 (K15919)	Glyoxylate and dicarboxylate metabolism (ko00630)
Unigene67243	1.05	1.02	Adenine phosphoribosyltransferase [EC:2.4.2.7] (K00759)	Purine metabolism (ko00230)
Unigene68035	10.71	9.84	Cytosine deaminase (K01485)	Pyrimidine metabolism (ko00240)
Unigene21052	-1.47	-2.88	Cysteine synthase A [EC:2.5.1.47] (K01738)	Cysteine and methionine metabolism (ko00270)
Unigene27404	2.66	9.16	high mobility group protein B1 (K10802)	Base excision repair (ko03410)
Unigene28613	1.93	3.49	Aldehyde dehydrogenase (NAD^+^) [EC:1.2.1.3] (K00128)	
Unigene63233	1.12	1.49	translation initiation factor eIF-5A (K03263)	
Unigene67559	-2.05	-6.44	cytochrome P450 [EC:1.14.-.-] (K00517)	
Unigene43786	-1.34	-5.79	cytochrome P450 [EC:1.14.-.-] (K00517)	
Unigene72576	1.60	1.66	cytochrome P450 [EC:1.14.-.-] (K00517)	
Unigene74562	-1.36	-3.55	E3 ubiquitin-protein ligase BRE1 [EC:6.3.2.19] (K10696)	
Unigene29179	-3.74	-9.39	E3 ubiquitin-protein ligase SHPRH (K15710)	
Unigene65959	1.26	1.47	[EC:2.7.1.-] (K00924)	
Unigene57553	1.05	3.55	ribonuclease T2 [EC:3.1.27.1] (K01166)	
Unigene25451	9.55	9.79	translation initiation factor IF-2 (K02519)	
Unigene27056	1.54	1.40	diacylglycerol kinase-like protein (K07029)	
Unigene13799	2.26	4.66	xyloglucan:xyloglucosyl transferase [EC:2.4.1.207] (K08235)	
Unigene70366	1.65	3.83	hypothetical protein (K09122)	
Unigene72849	2.96	8.74	aquaporin TIP (K09873)	
Unigene49577	-4.60	-4.50	histone H3 (K11253)	
Unigene19773	1.66	2.01	nucleolin (K11294)	
Unigene61235	-1.98	-2.25	WD repeat-containing protein 23 (K11801)	
Unigene26011	1.57	1.04	Niemann-Pick C1 protein (K12385)	
Unigene30461	4.75	3.88	beta-aspartyl-peptidase (threonine type) [EC:3.4.19.5] (K13051)	
Unigene15169	1.05	12.68	beta-mannan synthase [EC:2.4.1.32] (K13680)	
Unigene25344	2.29	1.60	solute carrier family 23 (nucleobase transporter) (K14611 )	
Unigene38865	2.16	4.03	serine carboxypeptidase-like clade IV [EC:3.4.16.-] (K16298)	

Analysis of the 14 DEGs related to energy metabolism revealed that the 11 DEGs related to photosynthesis in the chloroplasts were significantly down-regulated and the two DEGs correlated to oxidative phosphorylation in the mitochondria were up-regulated after PaWB *Phytoplasma* infection. The protein products of the genes involved in the photosynthesis pathway were all important components of photosynthetic complexes, including components of photosystem I (Unigene23994 and Unigene41021), photosystem II (Unigene57786, Unigene70434, Unigene73155, Unigene67411 and Unigene40507), F-type H^+^-transporting ATPase (Unigene 27161 and Unigene72265) and antenna proteins (Unigene57813 and Unigene65637). The analysis of the DEGs involved in energy metabolism indicated that infection by PaWB *Phytoplasma* influenced the energy metabolism of paulownia mainly via the suppression of photosynthesis.

Of the 14 DEGs associated with the translation process, five DEGs were up-regulated and nine DEGs were down-regulated after PaWB *Phytoplasma* infection. Of the five up-regulated DEGs, two were related to aminoacyl-tRNA biosynthesis (Unigene60861 and Unigene50004), one to cytoplasm ribosomes (Unigene44862) and two to plastid ribosomes (Unigene12998 and Unigene53304). Of the nine down-regulated DEGs, seven were related to chloroplast ribosomes (Unigene77, Unigene52586, Unigene24837, Unigene60918, Unigene68812, Unigene1219 and Unigene62803), one to cytoplasm ribosomes (Unigene37795) and one to RNA transport (Unigene74177). These results suggested that the translation process in chloroplasts was significantly suppressed after *Phytoplasma* infection, but the translation processes in the cytoplasm or mitochondria were neither significantly enhanced nor suppressed.

The DEGs involved in carbohydrate metabolism were mainly associated with cell wall biosynthesis and degradation. UDP-glucose 4-epimerase (Unigene65685), α-galactosidase (Unigene68516) and inositol oxygenase (Unigene23754) were involved in the biosynthesis of the nucleotide sugar precursors of cell-wall matrix polysaccharides. Cellulose synthase A (Unigene35494 and Unigene112), pectinesterase (Unigene36507 and Unigene67084) and beta-glucosidase (Unigene66203) were involved in the formation, modification and degradation of cell-wall matrix polysaccharides. Fructokinase (Unigene74438) was an important enzyme involved in metabolism of fructose, a sugar that accounts for half of the sucrose-derived carbon. 

Among the common DEGs in the two experimental groups, 12 DEGs were related to plant hormones. eight were involved in the biosynthesis of several hormones and four were correlated with hormone signal transduction. Phospholipase A2 (Unigene68742) and enoyl-CoA hydratase/ 3-hydroxyacyl-CoA dehydrogenase (Unigene6332) were possibly correlated with jasmonic acid biosynthesis. Isopentenyl diphosphate isomerase (*IPI*, Unigene1276), adenylate isopentenyltransferase (*IPT*, Unigene22539 and Unigene554) and cytokinin trans-hydroxylase CYP735A (Unigene73098) were involved in cytokinin biosynthesis. Farnesyl diphosphate synthase (Unigene10488) and xanthoxin dehydrogenase (Unigene4457) were related to abscisic acid biosynthesis. Phytochrome-interacting factor 3 (Unigene26784) and auxin influx carrier (Unigene74076) were involved in hormone signal transduction and delta24-sterol reductase (Unigene70471) and phospholipase C (Unigene28278) were correlated to hormone signal responses [19–21]. These results indicated that *Phytoplasma* infection significantly influenced the biosynthesis and signal transduction of several plant hormones, which may cause the symptoms seen in diseased plants.

The DGE results showed that five potential defense-related genes were induced after infection with PaWB *Phytoplasma*. Four of them encoded transcription factors and protein kinases, namely serine/threonine-protein kinase PBS1 (Unigene25741 and Unigene567), LRR receptor-like serine/ threonine-protein kinase (Unigene66077) and guanine nucleotide exchange factor (GEF) (Unigene22762). The remaining gene encoded L-ascorbate peroxidase (Unigene13928). Interestingly, two DEGs encoding (*S*)-2-hydroxy-acid oxidase (Unigene814) and nitric-oxide synthase (Unigene71586) were down-regulated after *Phytoplasma* infection. These results suggested that defense responses were induced but not completely and effectively activated to kill the *Phytoplasma* in the diseased plants.

### Comparison of DGE tag data with the qRT-PCR expression pattern

**Figure 6:**
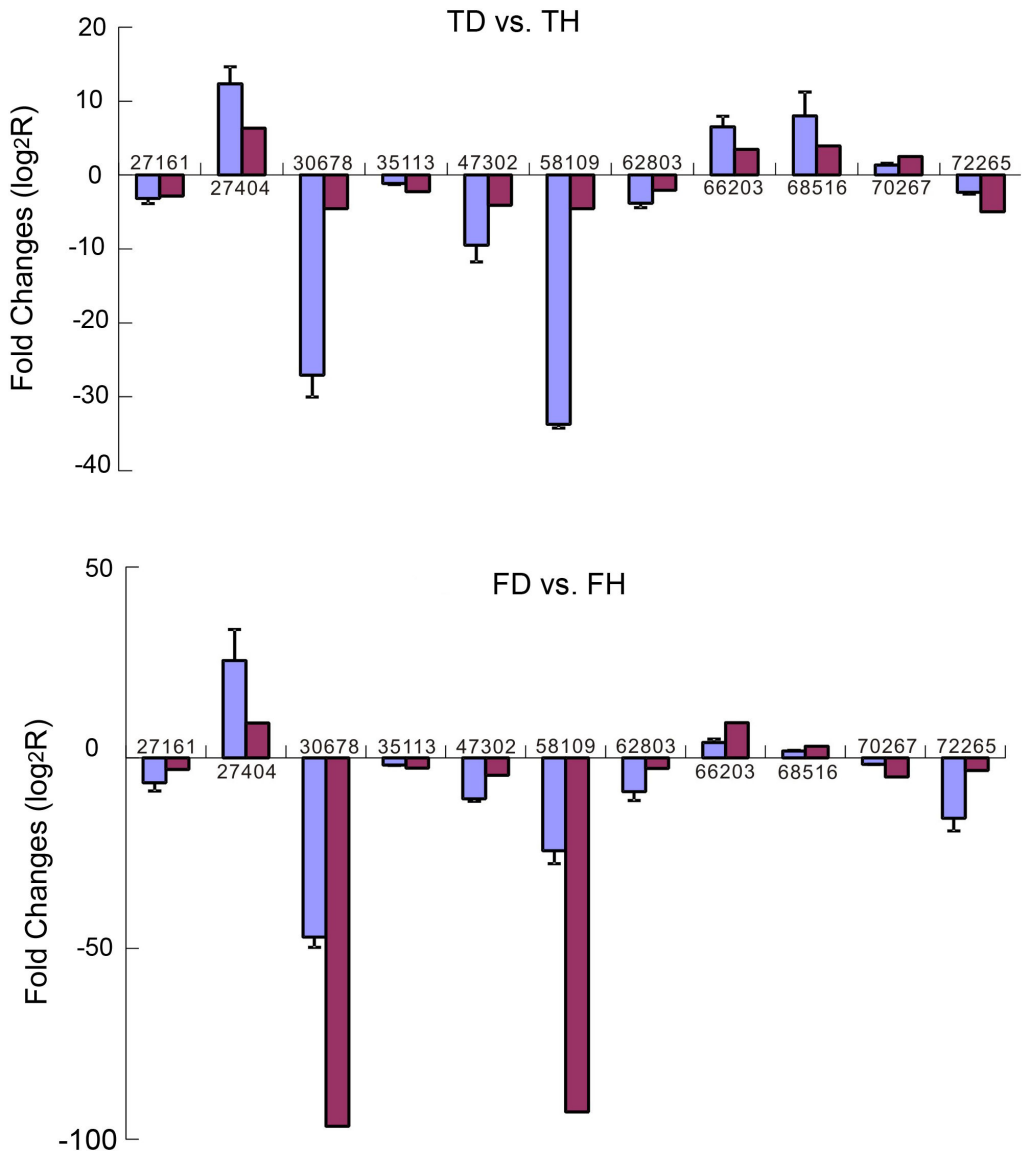
Validation of relative expression levels of the transcripts selected from the DGE analysis results using quantitative RT-PCR (qRT-PCR). Expression profiles of the seven selected genes were determined by qRT-PCR (Blue) and DGE (Red). The signal intensity of each transcript was normalized using actin. The predicted function of each gene is shown in Table 5. The error bars represent the standard deviations of the qRT-PCR signals from each of the three independent samples.

We used qRT-PCR to analyze the expressions of eleven selected genes in order to validate the gene expression differences revealed by the DGE experiments. The real-time PCR primers were shown in Table 5.The group consisted of three down-regulated genes in both the field grown group and tissue cultured group, i.e. Unigene 27161, Ungene72265, Unigene62803, Unigene 30678, Unigene 35113, Unigene 47302, Unigene 58109; two up-regulated genes Unigene66203, Ungene27404, Unigene68516; one gene up-regulated in the field grown group but down-regulated gene in the tissue cultured group and encoded the ABC transporter family protein (unigene 70267). The PCR experiments for each gene were performed using three biological replicates. The results obtained by qRT-PCR were consistent with the DGE analysis results (Figure 6), which suggested that the gene expression changes detected by DGE analysis were reliable.

**Table 5:** Primer sets used in the qRT-PCR analysis.

Predicted function of gene	Primer name	Nucleotide sequence (5′3′)
ATP synthase CF1 alpha subunit protein	27161F	TTGCAGAATTAGAAGCCTTTG
	27161R	GGAGCTGATTGGGATTGTTTA
ABC transporter family protein	70267F	GAGGCGTGTTAATGGTCAGT
	70267R	AGCCGATTGTAGTATCTTCTTGTA
ATP synthase delta chain, putative	72265F	CACAAGAACTTCATTCCTCCATT
	72265R	GACAGCCTCTTCACATCCCTC
Elongation factor EF-Tu [EC:3.6.5.3]	62803F	GGTCATGCCTGGTGATCGTG
	62803R	AGCACCTACAGTCTTCCCACCTT
High mobility group family	27404F	CCTCCTCTTCTTCGTCTTCTG
	27404R	CTGCTGGCTTCTTCTTGGTCT
Beta-glucosidase-related glycosidases	66203F	GTGGCTGATGCTCTGTTTGG
	66203R	CTTGCCCGGGTTTAGTTGTA
Cinnamyl-alcohol dehydrogenase [EC:1.1.1.195]	30678F	CTGCGCCAAACAGATTCTCAC
	30678R	CAACGGACGACGAAATGCTC
Ubiquitin carboxyl-terminal hydrolase L3	35113F	TCTCTCCACATTGTTTCTTTAGGG
	35113R	AGATTGTTGTTTGACTTGAGATGCT
Inorganic pyrophosphatase [EC:3.6.1.1]	47302F	AGATAGCAGACGAATGAATGTAGCC
	47302R	CGTAACAACCTTGCCAAAAATG
Acyl-CoA oxidase [EC:1.3.3.6]	58109F	TTTGAGCTTGCCCCGACAT
	58109R	GTTTACGCAGCAGGAGAGGAG
Methylsterol monooxygenase [EC:1.14.13.72]	68516F	ACATCGTGATACAGCCTTTTTG
	68516R	CCATCCGCTAATCTCCCATAA

## Discussion

Understanding the plant responses to pathogen infection requires a comprehensive evaluation of the changes in the gene expression profiles induced by pathogens. RNA-Seq and DGE techniques, based on next generation sequencing, are powerful methods that are used to study gene expression changes in plant hosts induced by pathogen infection, especially for plants where the genome sequence is not unavailable [22]. We used RNA-Seq combined with DGE to study the gene expression alterations in paulownia caused by PaWB *Phytoplasma* infection. To our knowledge, this is the first study on the gene expression profile of paulownia at the whole genome level. Our study provides comprehensive information on both paulownia gene expression and PaWB infection and provides a foundation for further study into paulownia and PaWB *Phytoplasma* infection. Our RNA-Seq analysis identified a total of 74,831 unigenes in paulownia and produced a database of unigene sequences for further analysis. Furthermore, DGE analysis of diseased and healthy cultured and field grown paulownia revealed that important biological processes were affected by PaWB *Phytoplasma* infection, such as plant hormone, photosynthesis and carbohydrate metabolism, which gave insights into the molecular mechanism behind the interaction between paulownia and PaWB *Phytoplasma*.

### Changes to hormone biosynthesis and their effect on plant symptoms

**Figure 7:**
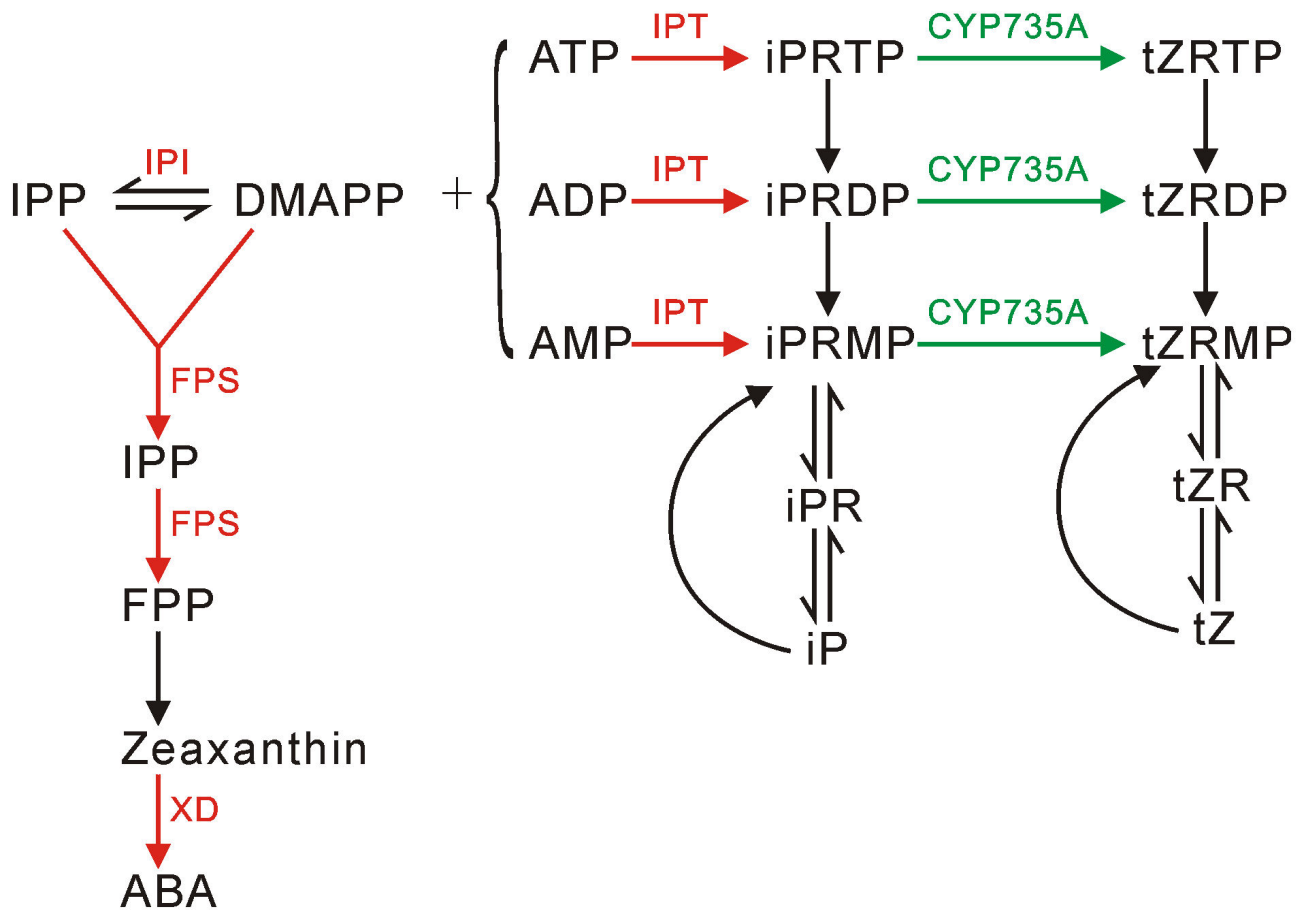
A model for cytokinin (CK) and abscisic acid (ABA) biosynthesis in paulownia infected with PaWB *Phytoplasma*. In plants, CKs are synthesized mainly through two pathways, namely cis-zeatin (cZ) biosynthesis and trans-zeatin (tZ) biosynthesis. Dimethylallyl diphosphate (DMAPP) is the common substrate for both pathways and is formed through isomerization of isopentenyl diphosphate (IPP), catalyzed by isopentenyl diphosphate isomerase (IPI).. Trans-zeatin biosynthesis, which is the main pathway for cytokinin synthesis in most plants, produces isopentenyladenine (iP)-type and tZ-type CKs. Adenylate isopentenyltransferases (IPT) catalyze the reaction between DMAPP and AMP/ADP/ATP to produce iP-nucleotides, which are then converted to tZ-nucleotides by cytokinin trans-hydroxylase (CYP735A). For ABA biosynthesis, farnesyl diphosphate synthase (FPS) catalyzes the synthesis of the isoprenoid precursors from both IPP and DMAPP and xanthoxin dehydrogenase (XD) catalyzes the last step in ABA formation. The up-regulated and down-regulated genes in paulownia after PaWB *Phytoplasma* infection are indicated in red and green, respectively. cZR: cZ riboside, GPP: geranyl diphosphate, FPP: farnesyl-diphosphate; iPRTP: iP riboside 5′-triphosphate; iPRDP: iP riboside 5′-diphosphate; iPRMP: iP riboside 5′-monophosphate; iPR: iP riboside; tZRTP: tZ riboside 5′-triphosphate; tZRDP: tZ riboside 5′-diphosphate; tZRMP: tZ riboside 5′-monophosphate; tZR: tZ riboside.

Plant hormones are thought to be involved in symptom formation by *Phytoplasma*, such as witches’-broom or dwarf symptoms [23,24]. It has been reported that increased cytokinin levels (CK) may induce witches’-broom or proliferation symptoms and that auxins had anti-*Phytoplasma* activity [25,26]. Our DGE analysis showed that several genes involved in the biosynthesis of cytokinin and abscisic acid (ABA) were influenced by PaWB *Phytoplasma* infection (Figure 7), which indicated that the typical symptoms of PaWB *Phytoplasma* infection might be due to changes in the expressions of CK and ABA-related genes.

After PaWB *Phytoplasma* infection, the expressions of paulownia genes encoding isopentenyl diphosphate isomerase (*IPI*, Unigene1276) and adenylate isopentenyltransferase (*IPT*, Unigene22539 and Unigene554) were enhanced, but cytokinin trans-hydroxylase CYP735A (Unigene73098) was suppressed. IPI is considered to be a checkpoint for isoprenoid biosynthesis and CK biosynthesis and IPT catalyzes the first and rate-limiting step of trans-zeatin (tZ) biosynthesis [27-30]. Up-regulation of paulownia genes encoding IPI and IPT may increase the concentration of CKs. Down-regulation of the paulownia gene encoding CYP735A would affect the conversion of isopentenyladenine (iP)-nucleotide to tZ-nucleotide. Our results for the genes involved in cytokinin biosynthesis suggested that the total CK content may have risen due to the increased expressions of paulownia IPT, caused by the PaWB *Phytoplasma*, and most CKs may exist as iP-type form. The expression of genes involved in auxin biosynthesis did not change significantly after PaWB infection in our study, which indicated that auxin content had neither dramatically increased nor decreased. The increased CK levels and the relatively constant auxin levels would result in an elevated cytokinin/auxin ratio, which may induce apical dominance and the formation of lateral buds, as suggested by previous investigations [31,32]. The increase in the number of lateral buds would result in witches’-broom symptoms. In conclusion, our investigation suggested that cytokinin might be a key phytohormone related to the formation of witches’-broom, which is the major symptom shown by paulownia when it is infected by PaWB *Phytoplasma*.

After PaWB *Phytoplasma* infection, the genes encoding farnesyl diphosphate synthase (*FPS*, unigene 10488) and xanthoxin dehydrogenase (XD) were up-regulated. FPS is considered a key enzyme in isoprenoid biosynthesis, which supplies carotenoid precursors for ABA biosynthesis, and XD catalyzes the last steps in the main ABA biosynthetic pathway [33-35]. The ABA content may increase as a result of the up-regulation of genes encoding FPS and XD. Previous studies demonstrated that ABA was a stress-related signaling molecule [36-38], which could inhibit stem elongation and shoot growth [39,40]. Therefore, the results suggest that the smaller leaves and shorter internode symptoms expressed after PaWB *Phytoplasma* infection may be associated with an increase in ABA. 

### Changes in cell wall metabolism and their effects on carbohydrate accumulation

Previous studies showed that the concentration and translocation of carbohydrates in plants were influenced by *Phytoplasma* infection [41-44]. Sugars and starch accumulated in the leaves of *Phytoplasma*-infected periwinkle, tobacco, corn and coconut palm [45,46]. Similarly, lethal yellowing *Phytoplasma* infection led to the accumulation of sugars and starch in the intermediate and upper leaves of coconut palms [47]. Therefore, *Phytoplasma* may alter the activity of specific enzymes involved in carbohydrate metabolism in infected plants in order to meet their requirements for energy, growth and spread [7]. Studies on grapevines infected by ‘Bois noir’ *Phytoplasma* suggested that BN *Phytoplasma* infection influenced the gene expression of enzymes involved in sucrose metabolism and callose deposition [7]. Our DGE analyses suggested that PaWB *Phytoplasma* infection may alter the expressions of a number of genes involved in cell wall biosynthesis and degradation and thus influence the carbohydrate metabolism of plants in order to satisfy their energy needs for growth and spread. 

Cellulose and pectin are the main components of plant cell wall polysaccharides and are formed by the polymerization of the nucleotidyl monosaccharide precursors [48]. In turn, the degradation of cellulose and pectin by specific enzymes generates disaccharides and monosaccharides. UDP-glucose 4-epimerase (Unigene65685), α-galactosidase (Unigene68516) and inositol oxygenase (Unigene23754) facilitate the biosynthesis of the nucleotide sugar precursors and cellulose synthase A (Unigene35494 and Unigene112) catalyzes the formation of cellulose [49,50]. Beta-glucosidase (Unigene66203) is a rate-limiting factor during the enzymatic hydrolysis of cellulose [51] and pectinesterase (Unigene36507 and Unigene67084) catalyzes the first step of pectin hydrolysis [52]. Fructokinase (Unigene74438) plays an important role in maintaining the flux of carbon towards cell wall nucleotidyl monosaccharide precursors, and the inhibition of fructokinase activity led to the accumulation of soluble neutral sugars and a decrease in hexose phosphates and UDP-glucose [53]. Our DGE results suggested that only the DEGs encoding fructokinase were down-regulated after *Phytoplasma* infection. The other above-mentioned DEGs were all up-regulated. Based on these results, we suggest that in diseased plants, both the formation of cellulose and the degradation of cellulose and pectin were enhanced, which might result in the accumulation of monosaccharides rather than nucleotidyl monosaccharides. 

### Changes in genes involved in photosynthesis

Previous studies revealed that genes encoding proteins involved in photosynthesis were down-regulated by biotic damage [54]. It has also been reported that *Phytoplasma* diseases caused a marked reduction in photosynthetic whole chain activity (mainly photosystem II activity) due to the loss of several thylakoid membrane proteins and a reduction in leaf soluble proteins [26]. In this study, 11 genes involved in the photosynthesis pathway were significantly down-regulated in the infected paulownia. These genes all encoded important photosynthetic complex components, including core protein PsaB (Unigene23994) and subunit II PsaD (Unigene41021) in photosystem I; core protein PsbA (Unigene57786), oxygen-evolving enhancer protein PsbP (Unigene70434), PsbQ (Unigene73155), 10 kDa protein PsbR (Unigene67411) and 22kDa protein PsbS (Unigene 40507) in photosystem II and subunit alpha (Unigene 27161), the delta subunit of F-type ATPase (Unigene72265), chlorophyll a/b binding protein LHCA2 (Unigene57813) and LHCA5 (Unigene65637) in light-harvesting complex I. In addition, seven genes associated with chloroplast ribosomes were significantly down-regulated in leaves with PaWB *Phytoplasma* infection, which indicated that the translation process in chloroplast ribosomes had been suppressed. These results suggest that PaWB *Phytoplasma* infection may result in a reduction in the gene expression of photosystem components at both the mRNA and the protein levels. Consequently, photosynthetic activity would be significantly repressed in infected leaves. In combination with the possible accumulation of monosaccharides, our results may support Christensen’s hypothesis that the accumulation of carbohydrates in source leaves led to photosynthesis feedback inhibition [23]. 

### Comparison between the field-grown condition and the tissue-cultured condition

As discussed above, there were many common DEGs and KEGG pathways after PaWB infection in both two groups. However, there were also differences between the two groups. First of all, the numbers of DEGs involved in the common KEGG pathways in field-grown group were usually larger than those in tissue-cultured group, and the expression change of the Unigenes in field-grown group were often bigger than those in tissue-cultured group. For example, in addition to the ten DEGs related to photosynthesis listed in Table 3, there were 10 more DEGs in field-grown group than in tissue-cultured group. In addition, several pathways associated with lipid metabolism in field grown condition were significantly inhibited after PaWB infection, and several important enzymes involved in those pathways were only down-regulated in field grown condition, such as acetyl-CoA acyltransferase (Unigene52895) and omega-3 fatty acid desaturase (Unigene46501). The inhibition of lipid metabolism might influence biosynthesis and function of downstream metabolites, and further disturb the normal condition of paulownia in field grown condition. These results indicated that the gene expression changes in field grown paulownia were more significant than those in plants that had been tissue cultured. A possible reason for this was that the environmental conditions present during tissue culturing were more stable than the environmental conditions that the field grown paulownia experienced. A complicated outdoor environment might interfere or strengthen the plant responses to pathogens. 

In summary, this investigation undertook transcriptomic analyses of paulownia using RNA-Seq and DGE for the first time. RNA-Seq analysis provided mRNA sequence information for paulownia and further DGE analysis on healthy paulownia and PaWB *Phytoplasma* infected paulownias under two growth conditions revealed potential biological processes related to disease symptoms and *Phytoplasma* survival. Although the molecular functions of some genes and their associated pathways remain largely unknown, this study provides insights into the paulownia responses to PaWB *Phytoplasma* infection at a transcriptomic level and will facilitate further investigations into the mechanisms behind the interactions between paulownia plants and *Phytoplasma*.

## Supporting Information

Figure S1 Components of the raw tags in each sample. The percentages of tags containing N, adaptors, the tags with a of copy number < 2 and the number of clean tags among the total raw tags are shown in parentheses.
(TIF)

Figure S2 Distribution of total clean tags (left) and distinct clean tags (right) in each sample. The numbers in square brackets indicate the range of copy numbers forof each tag category. and tThe percentages of corresponding tags percentages among the total clean tags are shown in parentheses. The numbers in square brackets indicate the range of copy numbers forof each tag category and the percentages of corresponding tags percentages among the distinct clean tags are shown in parentheses.
(TIF)

Table S1 Significantly enriched KEGG pathways in the FH vs the FD groups. (DOC)

Table S2 Significantly enriched KEGG pathways in the TH vs the TD groups. (DOC)
