# Supplementary figures and images for: Transcriptomic Analysis of Paulownia Infected by Paulownia Witches'-Broom Phytoplasma
Source: PLoS One. 2013 Oct 10;8(10):e77217. doi: 10.1371/journal.pone.0077217 (PMC3795066; doi:10.1371/journal.pone.0077217)

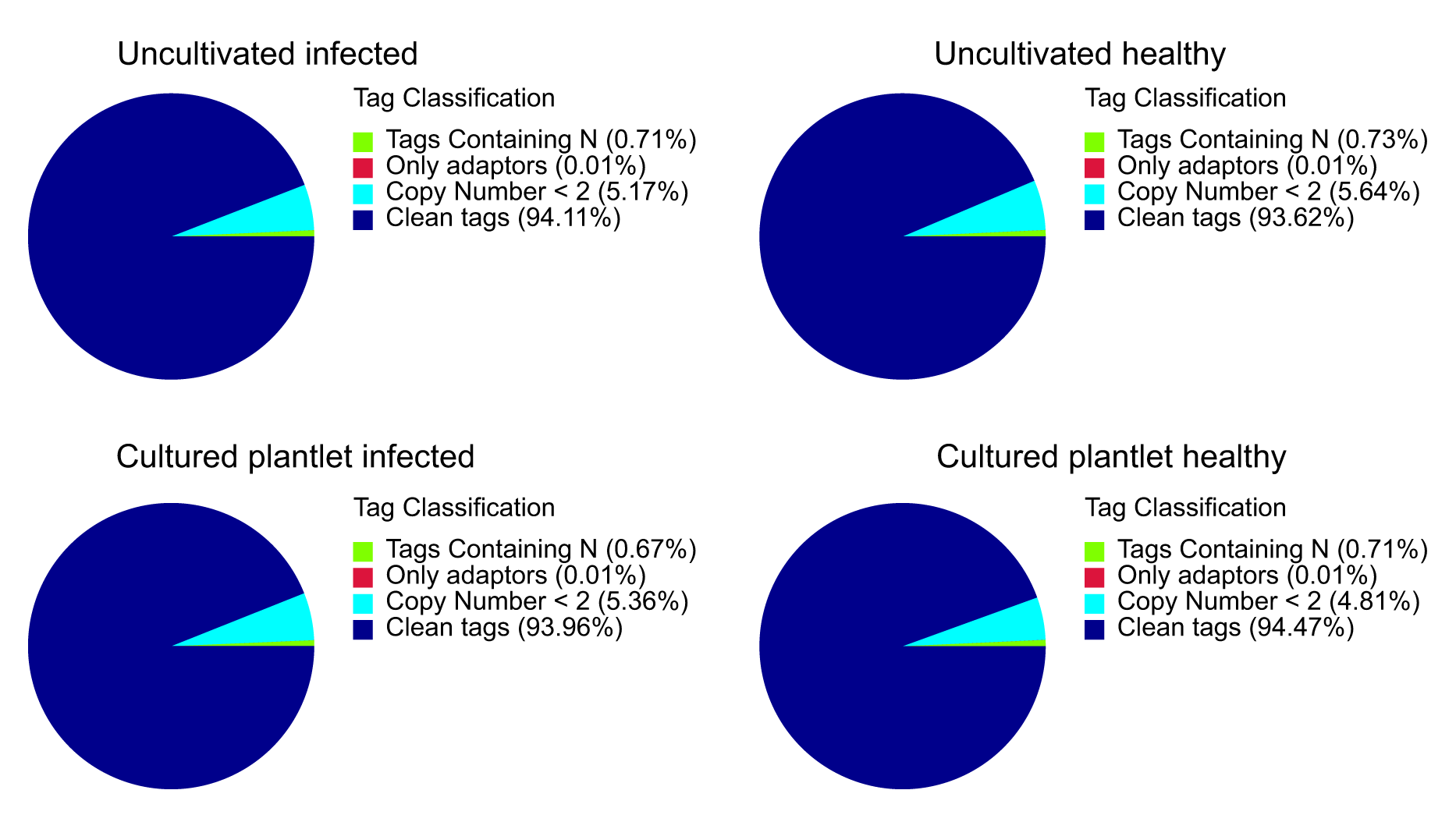

Supplement: Figure S1 — The percentages of tags containing N, adaptors, the tags with a of copy number < 2 and the number of clean tags among the total raw tags are shown in parentheses. (TIF) [file pone.0077217.s001.tif]

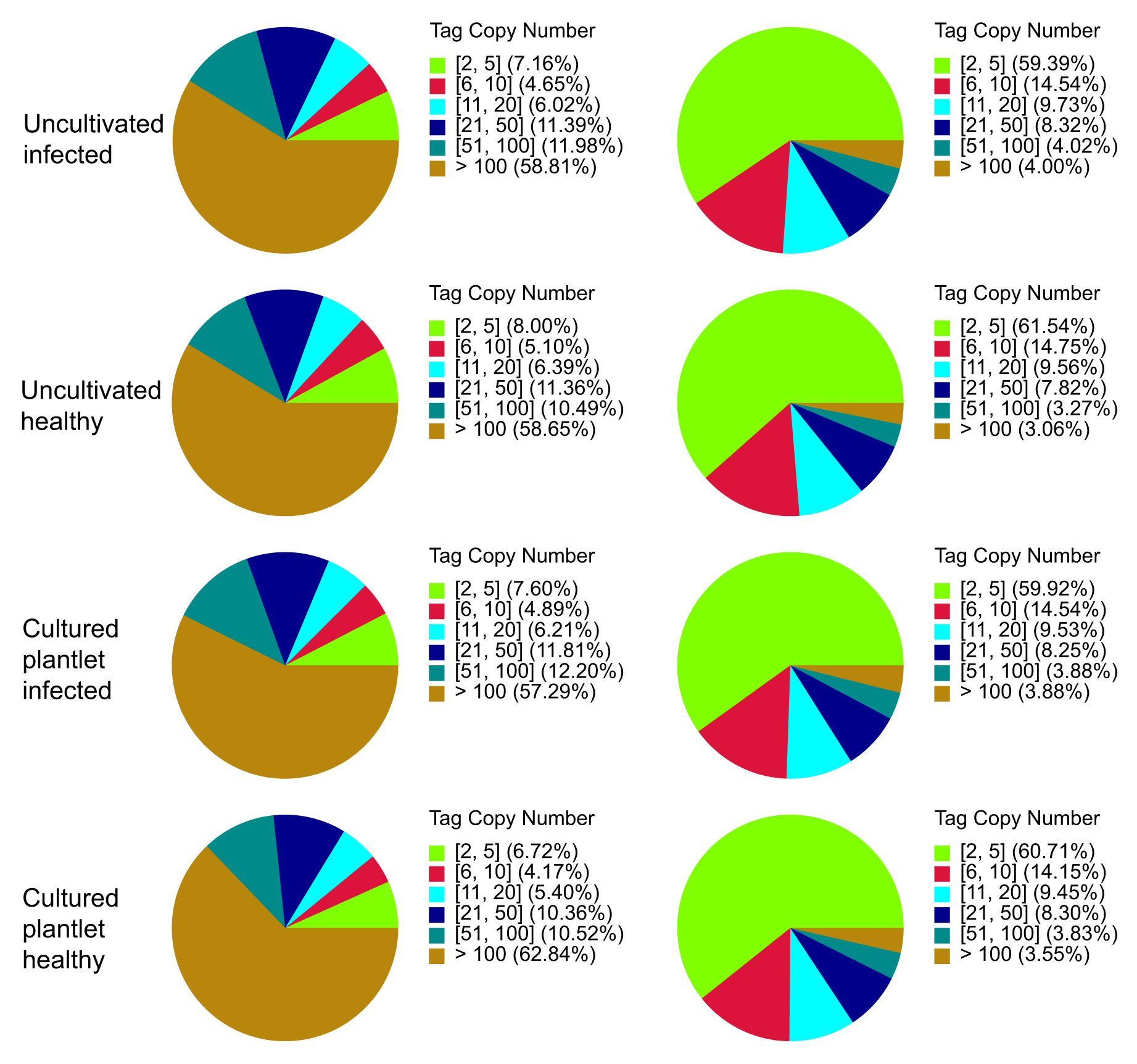

Supplement: Figure S2 — The numbers in square brackets indicate the range of copy numbers forof each tag category. and tThe percentages of corresponding tags percentages among the total clean tags are shown in parentheses. The numbers in square brackets indicate the range of copy numbers forof each tag category and the percentages of corresponding tags percentages among the distinct clean tags are shown in parentheses. (TIF) [file pone.0077217.s002.tif]
