# Supplementary material for: Transcriptomic Analysis of Paulownia Infected by Paulownia Witches'-Broom Phytoplasma
Source: PLoS One. 2013 Oct 10;8(10):e77217. doi: 10.1371/journal.pone.0077217 (PMC3795066; doi:10.1371/journal.pone.0077217)
Supplement: Table S2 — (DOC) [file pone.0077217.s004.doc]

Table S2. Significantly enriched KEGG pathways in THvsTD group.

| **Pathway ID** | **Pathway** | **Up-regulated genes** | **Down-regulated genes** | **P-value** | **KEGG Function Class** |
| --- | --- | --- | --- | --- | --- |
| ko00195 | Photosynthesis | 0 | 10 | 1.30E-05 | Metabolism; Energy Metabolism |
| ko00591 | Linoleic acid metabolism | 5 | 6 | 1.40E-05 | Metabolism; Lipid Metabolism |
| ko01100 | Metabolic pathways | 61 | 53 | 9.20E-05 | Metabolism; Overview |
| ko00592 | alpha-Linolenic acid metabolism | 6 | 6 | 3.00E-04 | Metabolism; Lipid Metabolism |
| ko00196 | Photosynthesis - antenna proteins | 0 | 5 | 3.90E-04 | Metabolism; Energy Metabolism |
| ko00980 | Metabolism of xenobiotics by cytochrome P450 | 5 | 4 | 1.30E-03 | Metabolism; Xenobiotics Biodegradation and Metabolism |
| ko01040 | Biosynthesis of unsaturated fatty acids | 3 | 5 | 3.30E-03 | Metabolism; Lipid Metabolism |
| ko00900 | Terpenoid backbone biosynthesis | 3 | 4 | 3.40E-03 | Metabolism; Metabolism of Terpenoids and Polyketides |
| ko00903 | Limonene and pinene degradation | 8 | 5 | 3.80E-03 | Metabolism; Metabolism of Terpenoids and Polyketides |
| ko00071 | Fatty acid metabolism | 5 | 5 | 4.00E-03 | Metabolism; Lipid Metabolism |
| ko00330 | Arginine and proline metabolism | 4 | 5 | 5.30E-03 | Metabolism; Amino Acid Metabolism |
| ko00941 | Flavonoid biosynthesis | 7 | 5 | 6.80E-03 | Metabolism; Biosynthesis of Other Secondary Metabolites |
| ko00590 | Arachidonic acid metabolism | 3 | 1 | 7.30E-03 | Metabolism; Lipid Metabolism |
| ko01070 | Biosynthesis of plant hormones | 16 | 15 | 8.30E-03 | Metabolism; Overview |
| ko00030 | Pentose phosphate pathway | 5 | 2 | 1.20E-02 | Metabolism; Carbohydrate Metabolism |
| ko03010 | Ribosome | 5 | 10 | 1.50E-02 | Genetic Information Processing; Translation |
| ko00906 | Carotenoid biosynthesis | 4 | 3 | 1.60E-02 | Metabolism; Metabolism of Terpenoids and Polyketides |
| ko01061 | Biosynthesis of phenylpropanoids | 14 | 11 | 1.70E-02 | Metabolism; Overview |
| ko00480 | Glutathione metabolism | 6 | 2 | 1.70E-02 | Metabolism; Metabolism of Other Amino Acids |
| ko00945 | Stilbenoid, diarylheptanoid and gingerol biosynthesis | 7 | 4 | 3.20E-02 | Metabolism; Biosynthesis of Other Secondary Metabolites |
| ko04146 | Peroxisome | 3 | 7 | 3.30E-02 | Cellular Processes; Transport and Catabolism |
| ko00940 | Phenylpropanoid biosynthesis | 12 | 4 | 3.40E-02 | Metabolism; Biosynthesis of Other Secondary Metabolites |
| ko00010 | Glycolysis / Gluconeogenesis | 8 | 4 | 4.50E-02 | Metabolism; Carbohydrate Metabolism |
